# Supplementary material for: Enhancing sensitivity and versatility of Tn5-based single cell omics
Source: Front Epigenet Epigenom. 2023 Nov 23;1:1245879. doi: 10.3389/freae.2023.1245879 (PMC13639286; doi:10.3389/freae.2023.1245879)
Supplement: Supplementary file 1 [file DataSheet1.pdf]

Supplementary Information

Enhancing sensitivity and versatility of Tn5-based single cell omics

Isabelle Seufert, Pooja Sant, Katharina Bauer, Afzal P. Syed, Karsten Rippe & Jan-Philipp Mallm

Inventory of Supplementary Information

Supplementary Figures

Figure S1. Quality assessment of scTurboATAC data

Figure S2. Transcription regulation analysis with scTurboATAC

Figure S3. Resolving cell types in PBMCs with scTurboATAC

Figure S4. Quality assessment of single cell multiome data

Supplementary Tables

Table S1. Ct values of standard curve for qPCR-based Tn5 activity test

Table S2. Ct values for qPCR activity measurements of Tn5 activity

Table S3. Sequencing metrics of scATAC and scTurboATAC data

Table S4. Cell numbers at varying unique fragments cutoffs for ATAC of MEF 6h INFβ

Table S5. Sequencing metrics of subsampled scATAC and scTurboATAC data

Table S6. Marker genes of hematopoietic cell types

Table S7. Sequencing metrics of scMultiome-ATAC in PBMCs

Table S8. Sequencing metrics of scMultiome-C&T in TF1 cell lines

Supplementary References

Supplementary Figures

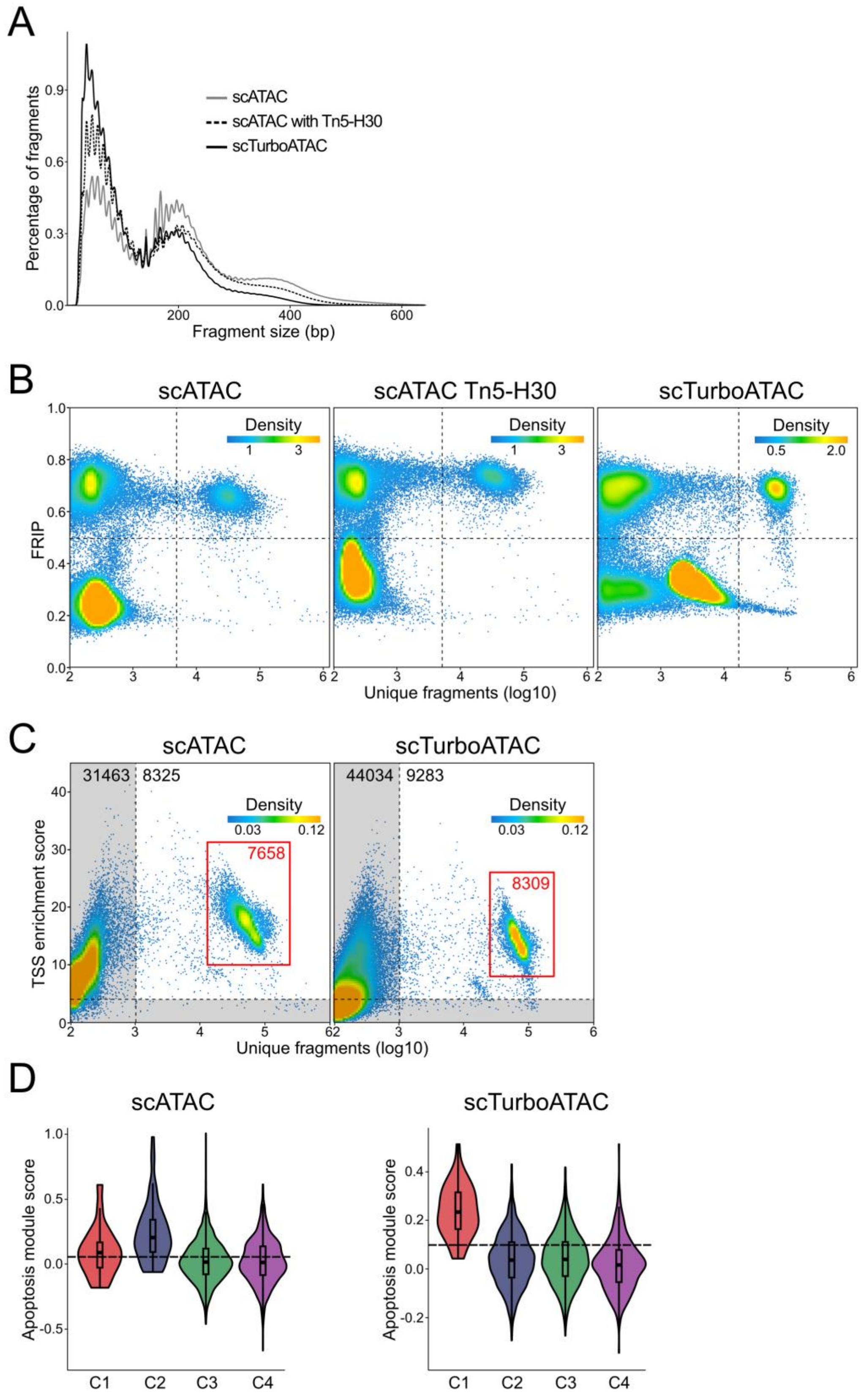

Fig. S1 Quality assessment of scTurboATAC data. (**A**) Fragment size distribution in

scATAC with Tn5-TXGv1.1 and Tn5-H30 versus scTurboATAC protocols for MEFs stimulated for 6h with IFNβ. (**B**) Fraction of reads in peaks (FRIP) against log10 number of unique fragments of cell barcodes in scATAC (left), scATAC with Tn5-H30 (middle) and scTurboATAC (right) of 6h IFNβ-stimulated MEFs. Colors of points reflect density of cell barcodes. (**C**) TSS enrichment score against log10 number of unique fragments of cell barcodes in scATAC with Tn5-TXGv2 (left) and scTurboATAC (right) for PBMCs. Colors of points reflect density of cell barcodes. Grey area marks low-quality cell barcodes. Red rectangle marks selected high-quality cells. (**D**) Apoptosis module score per cell in scATAC with Tn5-TXGv1.1 (left) and scTurboATAC (right) of 6h IFNβ-stimulated MEFs.

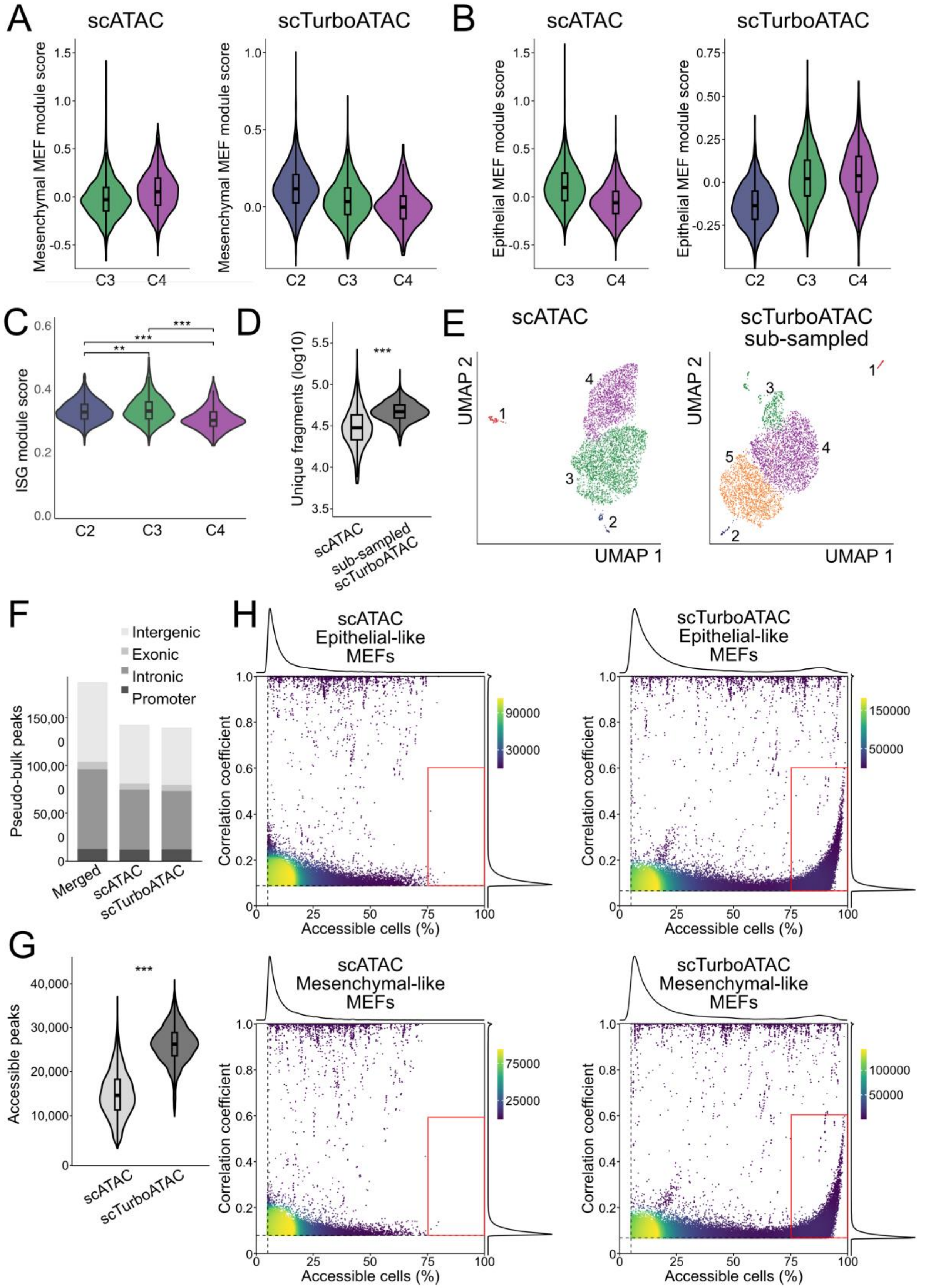

Fig. S2 Transcription regulation analysis with scTurboATAC. MEFs stimulated for 6h with IFNβ were studied. The scATAC data refer to the Tn5-TXGv1.1 enzyme. (**A**) Mesenchymal-like MEF module score per cell in scATAC and scTurboATAC. (**B**) Epithelial-like MEF module

score per cell in scATAC and scTurboATAC. (**C**) ISG module scores in scTurboATAC MEF clusters. P-values from Wilcoxon Rank Sum test (unpaired, two-sided) are indicated as *, P < 0.05; **, P < 0.01; ***, P < 0.001. (**D**) Number of unique fragments (log10) per cell. P-values from Student's t-test (two-sided, unpaired) are indicated as *, P < 0.05; **, P < 0.01; ***, P < 0.001. (**E**) UMAP embedding of 6h IFNβ-stimulated MEFs from scATAC (left) and subsampled scTurboATAC data (right). (**F**) Number of peaks from pseudo-bulk chromatin accessibility of scATAC, scTurboATAC and the merged peak set, which additionally included data from scATAC with Tn5-H30. (**G**) Number of accessible peaks detected per cell in scATAC and scTurboATAC using the merged peak set. P-values from Student's t-test (two-sided, unpaired) are indicated as *, P < 0.05; **, P < 0.01; ***, P < 0.001. (**H**) Correlation coefficient and percent accessible cells of co-accessible links from scATAC (left) and scTurboATAC (right). Colors of points reflect density of co-accessible links. Density curves of correlation coefficients and percent accessible cells are shown. Dashed lines represent background co-accessibility cutoff on correlation coefficient and minimal 5% cutoff on accessible cells. Red rectangle highlights major difference between scATAC and scTurboATAC.

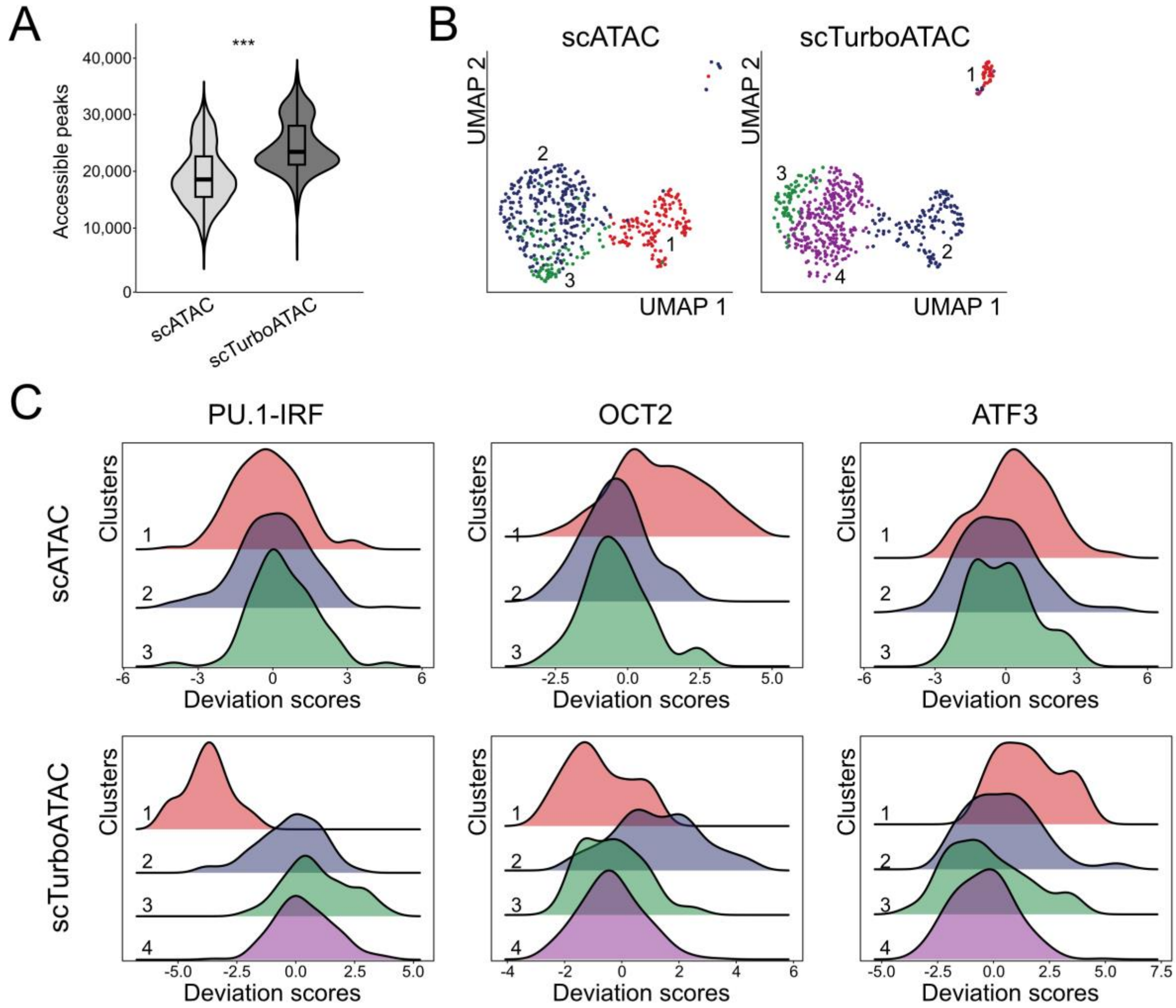

Fig. S3 Resolving cell types in PBMCs with scTurboATAC. The scATAC refers to data acquired with the Tn5-TXGv2 enzyme. (**A**) Number of accessible peaks per cell in scATAC and scTurboATAC using the merged peak set. P-valued from Student's t-test (two-sided, unpaired) are indicated as *, P < 0.05; **, P < 0.01; ***, P < 0.001. (**B**) Integrated UMAP embedding of B cells filtered from PBMC data set for scATAC and scTurboATAC. Integrated UMAP embeddings split by samples scATAC (left) and scTurboATAC (right) are shown. Cells are colored according to sample-specific k-nearest neighbor clusters. (**C**) Transcription factor motif deviation scores (non-imputed) in k-nearest neighbor clusters of scATAC (top) and scTurboATAC (bottom).

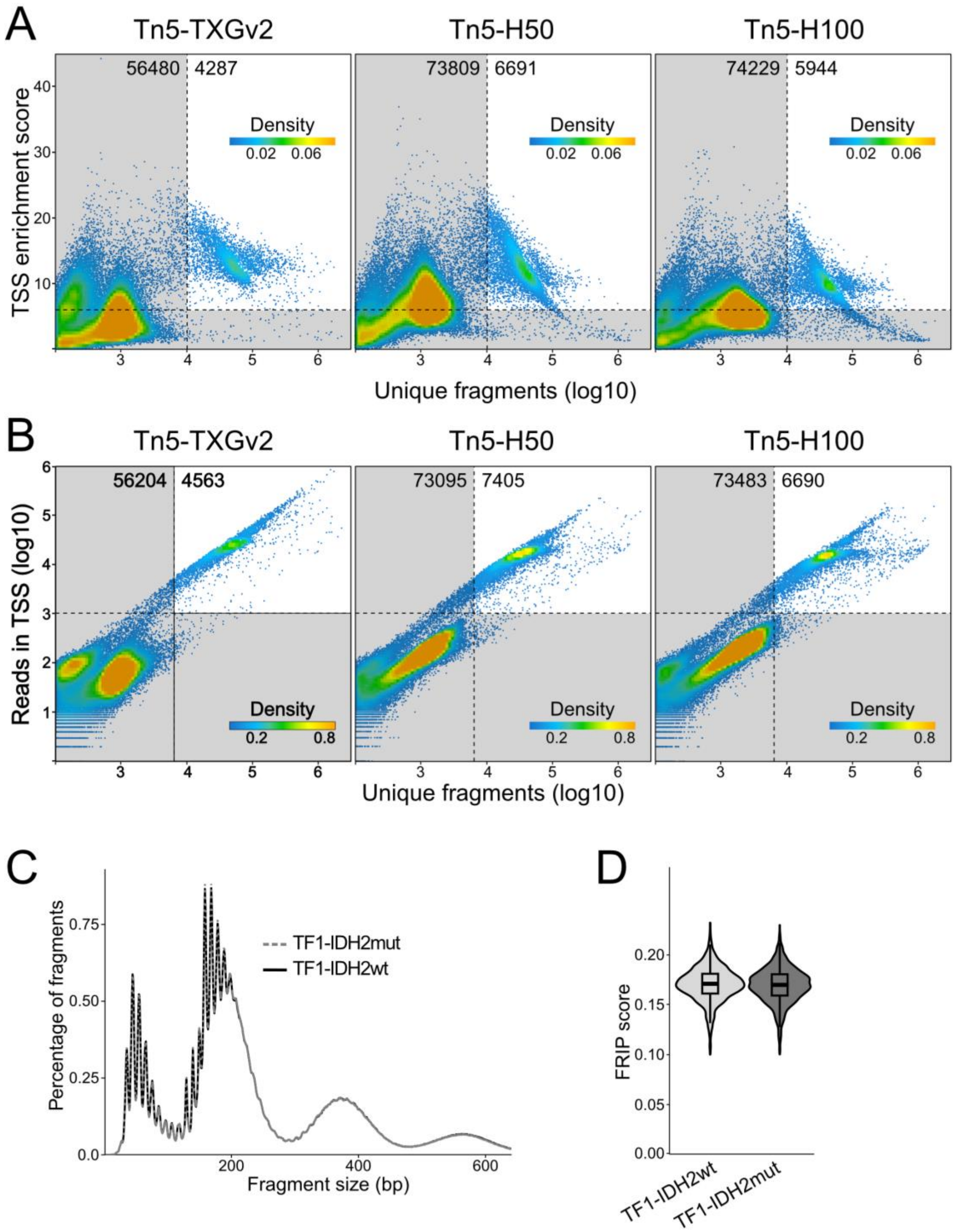

Fig. S4 Quality assessment of single cell multiome data. (**A**) TSS enrichment score over log10 number of unique fragments of cell barcodes in scMultiome-ATAC of PBMCs with the indicated Tn5 preparations. Coloring of points reflects the cell barcode density. Grey area marks low-quality cell barcodes. (**B**) Same as panel A for log10 number of reads in TSS over log10 number of unique fragments. (**C**) Fragment size distribution for scMultiome-C&T against H3K27me3 in TF1-IDH2wt and TF1-IDH2mut. (**D**) Fragments of reads in peaks (FRIP) scores for H3K27me3 per cell in TF1-IDH2wt and TF1-IDH2mut from the scMultiome-C&T analysis.

Supplementary Tables

Table S1. Ct values of standard curve for qPCR-based Tn5 activity test

Standard	Ct	Std. dev.
0	11.5216656	0.124669646
-0.3013	12.2683992	0.265657318
-0.60206	13.568965	0.4099452
-0.90309	14.5963147	0.375203068
-1.20412	15.0748687	0.374985728
-1.50515	16.5653429	0.297095495
**Slope**	-3.29	
**Efficiency**	101%	

Table S2. Ct values from qPCR activity measurements of Tn5 activity

**Activity measurement at different concentrations**			
**Tn5**	**Ct**	**Std. dev.**	**buffer**
H100	11.80788422	0.2202436	Tag buffer
H30	12.10263348	0.29840323	Tag buffer
H6	13.93965626	0.19020367	Tag buffer
TXGv1.1	12.35502243	0.34024468	Tag buffer
TXGv2	13.36461926	0.38053825	Tag buffer
**Activity measurement in different reaction buffers**			
**Tn5**	**Ct**	**Std. dev.**	**buffer**
H100	12.4007568	0.24680629	Tag buffer
H100	11.7715111	0.10708105	10X Buffer
Illumina	12.7094793	0.05563958	Tag buffer
Illumina	12.1429777	0.09835228	10X Buffer
TXGv2	13.5827904	0.16837424	Tag buffer
TXGv2	13.7061253	0.01677647	10X buffer

Table S3. Sequencing metrics of scATAC and scTurboATAC data

	MEF 6h IFNβ			PBMC	
	scATAC with Tn5-TXGv1.1	scATAC with Tn5-H30	scTurboATAC	scATAC with Tn5-TXGv2	scTurboATAC
Sequenced read pairs	827,356,762	1,221,969,310	1,304,430,940	1,466,934,091	1,468,516,560
Duplicates	70.1 %	75.8 %	45.9 %	66.6 %	52.2 %
Mapped read pairs	92.2 %	88.2 %	87.7 %	94.0 %	90.5 %
Fragments nucleosome-free regions	40.6 %	54.5 %	64.8 %	50.4 %	47.5 %

Table S4. Cell numbers at varying unique fragments cutoffs for ATAC of MEF 6h INFβ

Cutoff	scATAC with Tn5-TXGv1.1	scATAC with Tn5-H30	scTurboATAC
log10(nFrags) > 2 & log10 (nFrags) > 6.7	67,933	74,384	87,989
log10 (nFrags) > 4	5,019	5,733	7,105
% cell containing barcodes	7.4 %	7.7 %	8.1 %

Table S5. Sequencing metrics of subsampled scATAC and scTurboATAC data

	MEF 6h IFNβ		
	scATAC with Tn5-TXGv1.1	scATAC with Tn5-H30 subsampled	scTurboATAC subsampled
Sequenced read pairs	827,356,762	827,400,971	827,404,289
Duplicates	70.1 %	68.7 %	36.0 %
Mapped read pairs	92.2 %	88.2 %	87.7 %
Fragments nucleosome-free regions	40.6 %	54.9 %	65.7 %
Mean of unique fragments per cell	26,145	25,177	45,285

Table S6. Marker genes of hematopoietic cell types

Cell type	Marker gene	Reference
Naïve CD8+ T cells	CD8A, GZMB, CD3D, CD34, LEF1	(Terstappen et al., 1992; Uhlen et al., 2019)
Mature CD8+ T cells	CD8A, GZMB, CD3D, CD2	(Uhlen et al., 2019)
Naïve CD4+ T cells	CD4, IL7R, CCR7, CD3D, CD34, LEF1	(Terstappen et al., 1992; Uhlen et al., 2019)
Mature CD4+ T cells	CD4, IL7R, S100A4, CD3D, CD2, IL2, TNF, IL21	(Uhlen et al., 2019)
NK cells	GNLY, NKG7, GZMB, NCAM1, FCGR3A	(Uhlen et al., 2019; Karlsson et al., 2021)
B cells	MS4A1, CD79A, CD74, CD19	(Uhlen et al., 2019; Karlsson et al., 2021)
Progenitor cells	CD34, KIT, PROM1, PTPRC (negative marker), CD38 (negative marker)	(Terstappen et al., 1992; Monaco et al., 2019)
Classical monocytes	CD14, LYZ, CST3	(Uhlen et al., 2019)
Non-classical monocytes	FCGR3A, MS4A7	(Uhlen et al., 2019)
Dentritic cells	FCER1A, CST3, CD74	(Uhlen et al., 2019)
Basophils	ENPP3, CD69, CCR3, PTGDR2	(Monaco et al., 2019; Uhlen et al., 2019)
Neutrophils	CEACAM8, ITGAM, FCGR3B, PTPRC	(Uhlen et al., 2019)(Monaco et al., 2019)
Eosinophils	EPX, RNASE2, CLC, SIGLEC8	(Uhlen et al., 2019)
Erythrocytes	HBA2, HBB, HBA1, SLC4A1, EPB41	(Karlsson et al., 2021)
Platelets	PPBP, ITGA2B, PF4	(Shattil et al., 1985; Poncz et al., 1987; Majumdar et al., 1991)

Table S7. Sequencing metrics of scMultiome-ATAC analysis in PBMCs

	Tn5-TXGv2	Tn5-H50	Tn5-H100
Sequenced ATAC read pairs	1,304,897,617	1,431,221,793	1,444,079,941
Sequenced RNA read pairs	714,778,267	644,951,970	716,455,503
Duplicates ATAC	68.5 %	67.2 %	57.3 %
Duplicates RNA	92.3 %	91.8 %	92.2 %
Mapped ATAC read pairs	92.5 %	91.0 %	91.2 %
Mapped RNA read pairs	61.4 %	53.1 %	51.8 %

Table S8. Sequencing metrics of scMultiome-C&T in TF1 cell lines

	**TF1-IDH2wt**	**TF1-IDH2mut**
Sequenced H3K27me3 read pairs	486,259,010	468,044,936
Sequenced RNA read pairs	42,673,800	36,916,878
Duplicates H3K27me3	96.6 %	96.6 %
Duplicates RNA	27.0 %	29.5 %
Mapped H3K27me3 read pairs	84.1 %	83.5 %
Mapped RNA read pairs	68.5 %	68.5 %

Supplementary References

Karlsson, M., Zhang, C., Mear, L., Zhong, W., Digre, A., Katona, B., et al. (2021). A single-cell type transcriptomics map of human tissues. *Sci Adv* 7(31). doi: 10.1126/sciadv.abh2169.

Majumdar, S., Gonder, D., Koutsis, B., and Poncz, M. (1991). Characterization of the human beta-thromboglobulin gene. Comparison with the gene for platelet factor 4. *Journal of Biological Chemistry* 266(9), 5785-5789. doi: 10.1016/s0021-9258(19)67665-9.

Monaco, G., Lee, B., Xu, W., Mustafah, S., Hwang, Y.Y., Carre, C., et al. (2019). RNA-Seq Signatures Normalized by mRNA Abundance Allow Absolute Deconvolution of Human Immune Cell Types. *Cell Rep* 26(6), 1627-1640 e1627. doi: 10.1016/j.celrep.2019.01.041.

Poncz, M., Surrey, S., LaRocco, P., Weiss, M.J., Rappaport, E.F., Conway, T.M., et al. (1987). Cloning and characterization of platelet factor 4 cDNA derived from a human erythroleukemic cell line. *Blood* 69(1), 219-223. doi: 10.1182/blood.V69.1.219.219.

Shattil, S.J., Hoxie, J.A., Cunningham, M., and Brass, L.F. (1985). Changes in the platelet membrane glycoprotein IIb.IIIa complex during platelet activation. *J Biol Chem* 260(20), 11107-11114.

Terstappen, L.W., Huang, S., and Picker, L.J. (1992). Flow cytometric assessment of human T-cell differentiation in thymus and bone marrow. *Blood* 79(3), 666-677. doi: 10.1182/blood.V79.3.666.666.

Uhlen, M., Karlsson, M.J., Zhong, W., Tebani, A., Pou, C., Mikes, J., et al. (2019). A genome-wide transcriptomic analysis of protein-coding genes in human blood cells. *Science* 366(6472). doi: 10.1126/science.aax9198.
